# The 9-1-1 checkpoint clamp stimulates DNA resection by Dna2-Sgs1 and Exo1

**DOI:** 10.1093/nar/gku746

**Published:** 2014-08-13

**Authors:** Greg H.P. Ngo, Lata Balakrishnan, Marion Dubarry, Judith L. Campbell, David Lydall

**Affiliations:** 1 Institute for Cell and Molecular Biosciences (ICaMB), Medical School, Newcastle University, Newcastle upon Tyne, NE2 4HH, UK; 2 Department of Biochemistry and Biophysics, University of Rochester School of Medicine and Dentistry, Rochester, NY 14642, USA; 3 Divisions of Biology and Chemistry, Caltech, Braun Laboratories, Pasadena, CA 91125, USA

## Abstract

Single-stranded DNA (ssDNA) at DNA ends is an important regulator of the DNA damage response. Resection, the generation of ssDNA, affects DNA damage checkpoint activation, DNA repair pathway choice, ssDNA-associated mutation and replication fork stability. In eukaryotes, extensive DNA resection requires the nuclease Exo1 and nuclease/helicase pair: Dna2 and Sgs1^BLM^. How Exo1 and Dna2-Sgs1^BLM^ coordinate during resection remains poorly understood. The DNA damage checkpoint clamp (the 9-1-1 complex) has been reported to play an important role in stimulating resection but the exact mechanism remains unclear. Here we show that the human 9-1-1 complex enhances the cleavage of DNA by both DNA2 and EXO1 *in vitro*, showing that the resection-stimulatory role of the 9-1-1 complex is direct. We also show that in *Saccharomyces cerevisiae*, the 9-1-1 complex promotes both Dna2-Sgs1 and Exo1-dependent resection in response to uncapped telomeres. Our results suggest that the 9-1-1 complex facilitates resection by recruiting both Dna2-Sgs1 and Exo1 to sites of resection. This activity of the 9-1-1 complex in supporting resection is strongly inhibited by the checkpoint adaptor Rad9^53BP1^. Our results provide important mechanistic insights into how DNA resection is regulated by checkpoint proteins and have implications for genome stability in eukaryotes.

## INTRODUCTION

DNA resection, the nucleolytic degradation of one strand of DNA ends, has emerged as an important regulator of the DNA damage response (1,2). The substrates for resection, DNA ends, arise at uncapped telomeres, DNA double strand breaks (DSBs) and stalled replication forks. DNA resection generates single-stranded DNA (ssDNA) to trigger and maintain DNA damage checkpoint signaling, which induces cell cycle arrest, senescence (permanent cell cycle arrest) or apoptosis (cell death) (3,4). Furthermore, the extent of DNA resection affects DNA repair pathway choice by either homology directed repair or non-homologous end joining (2,5). Extensive DNA resection can also be harmful by increasing ssDNA-associated cancer-inducing mutation clusters (6–8). Furthermore, unregulated resection activities degrade stalled replication forks or dysfunctional telomeres (9–13). Thus proper regulation of DNA resection has important consequences on genome stability and cell viability.

In eukaryotes, DNA resection is carried out in two distinct steps (1,2). In the first step, MRX^MRN^ and Sae2^CtIP^ initiate the reaction by generating a short 3′ ssDNA overhang (14,15). In the second step, the nuclease Exo1 and/or nuclease/helicase pair: Dna2 and Sgs1^BLM^ carry out extensive DNA resection of up to 30 kb (9,14,15). Recent studies suggest that initiation of DNA resection is tightly regulated by post-translational modifications (phosphorylation, acetylation and ubiquitination) of MRX^MRN^ and Sae2^CtIP^ in budding yeast and higher eukaryotes (16–19). However, how Exo1 and Dna2-Sgs1 activities are coordinated to regulate extensive DNA resection remains poorly understood.

The DNA damage checkpoint stimulates signal transduction cascades that not only regulate cell cycle progression but also DNA repair (3,20). Several DNA damage checkpoint proteins play important, yet complex roles in regulating DNA resection. In budding yeast, Rad9^53BP1^ inhibits DNA resection through a mechanism largely independent of its role in activating checkpoint effector Rad53^CHK2^, but instead relies on its ability to bind to chromatin (21–24). 53BP1, the mammalian homolog of Rad9^53BP1^, also inhibits DNA resection (25). On the other hand, the 9-1-1 checkpoint complex promotes DNA resection at uncapped telomeres, DNA DSBs and stalled replication forks, but shows no nuclease activity *in vitro* and the biochemical mechanism of control remains poorly understood (26–31).

To better understand how the 9-1-1 complex promotes resection, we have used complementary biochemical and genetic techniques with human proteins and yeast cells. We show that the human 9-1-1 complex promotes DNA resection by stimulating the DNA binding and nucleolytic activity of both DNA2 and EXO1 *in vitro*. These roles are physiologically relevant and conserved, since in *Saccharomyces cerevisiae*, 9-1-1 mutants are defective in both Dna2-Sgs1 and Exo1-dependent resection. The activity of the 9-1-1 complex in supporting resection is strongly inhibited by Rad9^53BP1^. Together these data illuminate the role of the 9-1-1 complex in promoting extensive DNA resection and illustrate its role in the complex network that carefully regulates the response to damaged DNA ends.

## MATERIALS AND METHODS

### Yeast techniques

All experiments were performed on *S. cerevisiae*. We used the W303 genetic background strains (Supplementary Tables S1 and S2). Yeast strains with *cdc13-1 cdc15-2 bar1* mutations were arrested in G1 with alpha-factor at 23°C and released into 36°C to induce telomere uncapping. Cell cycle positions were scored using 4′,6-diamidino-2-phenylindole (DAPI) staining on a Nikon Eclipse 50i microscope. Quantitative amplification of ssDNA (QAOS) analyses were carried out as previously described (32,33). Chromatin immunoprecipitation (ChIP) were performed as previously described (34).

### Immunoprecipitation

Yeast pellets were lysed in IP buffer (pH7.5, 20-mM HEPES, 140-mM NaCl, 1-mM ethylenediaminetetraacetic acid (EDTA), 1% Triton X 100, 0.1% sodium deoxycholate, 2-mM phenylmethylsulfonyl fluoride (PMSF) and protease inhibitor cocktail) by bead beating. The cell lysates were incubated with anti HA (ab9110, Abcam) or anti Myc (ab32, Abcam) antibodies for 1 h and protein G Dynabeads (Invitrogen) were added before incubation on a wheel at 4°C overnight. The immunoprecipitates were washed four times in IP buffer, boiled in laemmli buffer and subjected to western blot analyses.

### Yeast two-hybrid

Genes of interest were polymerase chain reaction (PCR) amplified and cloned in yeast by recombinational cloning. The Gal4 activation domain (prey AD-X; pMB29) vector or Gal4 DNA binding domain (bait DB-Y; pMB27) vector (kind gifts from Mike Boxem) was linearized with SmaI and co-transformed into yeast with the PCR fragment of interest (see Supplementary Table S4). Typically, vector control gave <10 colonies whereas co-transformation with PCR product gave >1000 colonies. *cdc13-1* reporter strains DLY7451 and DLY7452 (derived from Y8800 and Y8930) (35) transformed with bait and prey plasmids were selected on SC-Trp or SC-Leu plates, respectively. Five colonies were pooled in SC-Trp or SC-Leu liquid media. Strains were mated in yeast extract peptone dextrose (YPD) (+ adenine) liquid culture to construct a set of diploid two-hybrid combination reporter strains. These diploid strains were cultivated to saturation in SC-Leu-Trp media before spotted at 23, 25, 26, 27, 28 and 29ºC for 4–7 days on the following plates: (i) SC-Leu-Trp – Cell growth control; (ii) SC-His-Leu-Trp – yeast two-hybrid (Y2H) interaction; (iii) SC-Ade-Leu-Trp – Y2H interaction; (iv) SC-His-Leu + 1mg/l CHX – Auto-activator detection and (v) SC-Ade-Leu + 1mg/l CHX – Auto-activator detection. Oligonucleotides used in two-hybrid experiments are listed in Supplementary Table S4.

### Purified human proteins

Recombinant human DNA2 was over-expressed using pFastBac HTc vector in baculovirus High Five (H5) cells and purified using a C-terminal FLAG tag as previously described (36). Rad9-Rad1-Hus1 (9-1-1) and RAD17-RFC were expressed in H5 insect cells and isolated as previously described (37,38). Proliferating cell nuclear antigen (PCNA) was expressed and purified as previously described (39). EXO1 was expressed and purified as previously described (40,41).

### Nuclease assays

Five fmol of substrate (flap, fork and 3′ ssDNA tail overhang) were incubated with various concentrations of DNA2, EXO1, 9-1-1 and PCNA in a reaction volume of 20 μl at 37°C for 10 min. For DNA2 nuclease assays, the reaction buffer consisted of 50-mM Tris HCl (pH 8.0), 2-mM dithiothreitol, 30-mM NaCl, 0.1-mg/ml bovine serum albumin, 4-mM MgCl2 and 2-mM adenosine triphosphate (ATP). For EXO1 nuclease assays, the reaction buffer consisted of 40-mM Tris-HCl (pH 7.6), 2-mM glutathione, 0.1-mg/ml bovine serum albumin, 10-mM MgCl2 and 3-mM ATP. The reactions were terminated using 2 X termination dye (90% formamide (v/v), 10-mM EDTA, 0.01% bromophenol blue and 0.01% xylene cyanol). After termination, samples were heated at 95°C for 5 min and 20 μl of reaction was loaded onto a denaturing gel (8-M urea)/18% polyacrylamide gel and electrophoresed for 1 h and 30 min at 80 W. Each experiment was performed at least in triplicate, and representative gels are shown in the figures. Gels were analyzed as previously described (42).

### Nuclease assay substrates

Synthetic oligonucleotides were synthesized by Integrated DNA Technologies and subjected to 3′-end labeling as previously described (43). Oligonucleotide sequences are listed in Supplementary Table S3. The downstream and upstream primer sequences are listed 5′ to 3′ and the template sequences are listed 3′ to 5′ to facilitate visual alignment. Substrates were created by annealing oligonucleotides as listed below: Flap Substrate: U1:D1:T1; Fork Substrate: D1:T1; double-stranded DNA (dsDNA) Substrate: D2:T2; 3′ overhang substrate: D3: T3.

## RESULTS

### Human 9-1-1 complex stimulates DNA2 and EXO1 cleavage activity

**Figure 1.**
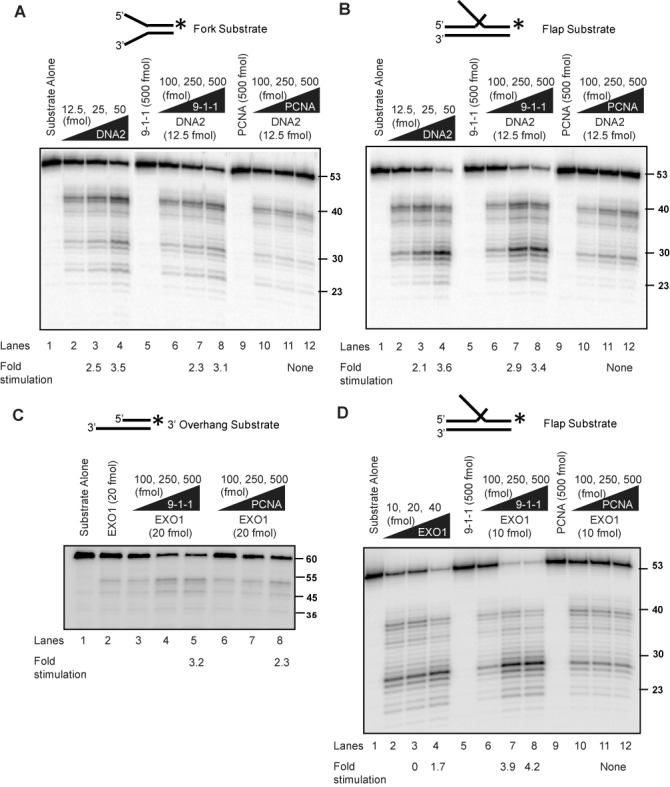
Human 9-1-1 complex stimulates DNA2 and EXO1 activities. (**A, B**) DNA2 cleavage activities on a fork (A) and a flap (B) substrate in the presence of the 9-1-1 complex or PCNA. Labeling of the substrate at the 3′ end allows the determination of the final products of the reaction. Products are analyzed on alkaline gels as described in the Materials and Methods section. (**C, D**) EXO1 cleavage activities on a 3′overhang (C) and a flap (D) structure in the presence of 9-1-1 or PCNA. Substrates used for each panel of the experiment are depicted on the top of the gel with the asterisk indicating the site of the ^32^P label. The flap is 30 nt long. The labeled strand in panel (C) is 60 nt long.

Rad24, the checkpoint sliding clamp (9-1-1) loader, was shown to promote resection *in vivo* nearly 20 years ago (26). Subsequent studies show that the 9-1-1 sliding clamp, itself, also promotes extensive telomere resection (23,24), but the initial model that Rad17, a component of the sliding clamp was a nuclease, has proven to be incorrect (28). Therefore we tested an alternative hypothesis that the 9-1-1 sliding clamp increases the activity of nucleases important for extensive DNA resection. Using recombinant human proteins, we examined the effect of 9-1-1 on DNA2 and EXO1 activity, the two nucleases involved in long-range resection. We first tested the activity of DNA2 on a fork structure, which mimics the unwound DNA substrate acted on by DNA2 during DNA resection (Figure 1A). Since the substrate is linear, the 9-1-1 toroidal clamp can slide onto the DNA in the absence of the RAD17/RFC clamp loader. DNA was labeled at the 3′ end in order to be able to detect all products formed (44). We found that DNA2 cleaved this substrate efficiently (Figure 1A, lanes 2–4). Importantly, we found that the 9-1-1 complex stimulated the cleavage of the linear substrate by DNA2 (Figure 1A; compare lane 2 with lanes 6–8). The stimulation of DNA2 cleavage was specific to the 9-1-1 complex, as addition of the related DNA replication clamp PCNA, which has a similar toroidal structure to the 9-1-1 complex, failed to stimulate the nuclease reaction (Figure 1A; compare lane 2 with lanes 10–12). The 9-1-1 complex or PCNA alone showed no nuclease or helicase activities (Figure 1A, lanes 5 and 9, and Supplementary Figure S1A). We conclude that 9-1-1 stimulates DNA2 nuclease activity on resection-mimic-substrates *in vitro*.

We next tested stimulation of the activity of DNA2 on a double-flap structure, which is a preferred substrate of DNA2 at replication forks (Figure 1B) (42). Importantly, similar to the result on the fork structure, addition of the 9-1-1 complex (compare lane 2 with lanes 6–8), but not PCNA (lanes 10–12) stimulated the DNA2 nuclease activity. Furthermore, we found that the 9-1-1 complex did not stimulate DNA2 degradation of a fully duplex DNA substrate or a gap substrate (Supplementary Figure S1B and C), suggesting 9-1-1 increases the activity of DNA2 on unwound ssDNA ends. These data suggest that 9-1-1 only stimulates the nuclease activity and not the helicase function of DNA2, since we did not observe any unwinding and subsequent cleavage of the dsDNA substrate (Supplementary Figure S1B). We conclude that 9-1-1 directly stimulates DNA2 cleavage activity. The effect of 9-1-1 is different to other stimulators of DNA2-Sgs1/BLM resection pathway (MRX/MRN and Top3-Rmi1), which do not stimulate DNA2 cleavage activity in the absence of Sgs1/BLM (45,46). The observation that 9-1-1 did not affect the cleavage pattern (Figure 1A and B) but lowered the concentration of DNA2 required for cleavage suggests that 9-1-1 may increase the effective local concentration of DNA2 at the substrate.

We next examined whether 9-1-1 affects EXO1 activity. We used dsDNA substrate with a 3′ tail, which mimics the structure formed during DNA resection. Use of a shorter substrate compared to the traditionally longer substrates used in resection assays (45) allowed us to probe 9-1-1 specific changes in the cleavage patterns of EXO1. We found that EXO1 cleaved this substrate inefficiently (Figure 1C; compare lanes 1 and 2). Interestingly, titrating in increasing amounts of 9-1-1 to the reaction resulted in increased cleavage of the substrate by EXO1 (Figure 1C; compare lane 2 with lanes 4 and 5). We found that PCNA also stimulated EXO1 cleavage activity (Figure 1C; compare lane 2 with lanes 7 and 8), as has been reported recently (47). As EXO1 also has flap endonuclease activity, we tested stimulation of the activity of EXO1 on a double-flap structure (Figure 1D). Importantly, similar to the result on the tailed structure, addition of the 9-1-1 complex (compare lane 2 with lanes 7 and 8) stimulated EXO1 activity. One interesting difference was that PCNA did not stimulate EXO1 on this substrate (Figure 1D, lanes 11 and 12). The observation that 9-1-1 did not affect the cleavage pattern of EXO1, but simply lowered the concentration of EXO1 required for cleavage of these substrates, suggests that 9-1-1 may increase the effective local concentration of EXO1 at the substrate. We conclude that the 9-1-1 complex promotes resection by directly stimulating DNA2 and EXO1 nuclease activities.

### Human 9-1-1 complex loads onto DNA to stimulate nuclease activity

**Figure 2.**
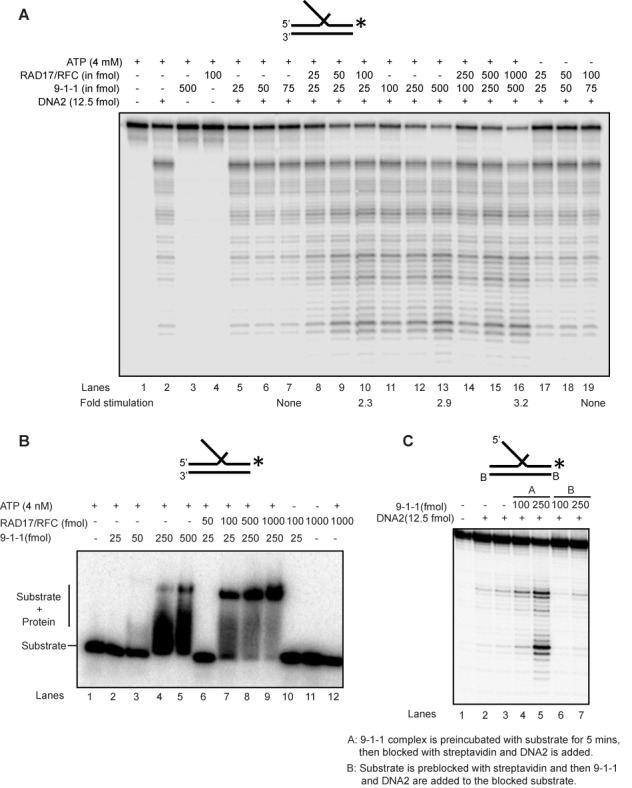
Human 9-1-1 complex loads onto DNA to stimulate nuclease activity. (**A**) DNA2 cleavage activity on a 5′ flap substrate in the presence of 9-1-1 and RAD17/RFC. (**B**) Binding efficiency of 9-1-1 (low and high concentration) in the presence of the clamp loader RAD17/RFC on a 5′ flap substrate. (**C**) DNA2 nuclease activity in the presence of the 9-1-1 complex on a substrate containing blocked template ends and free 5′ flap. Substrates used for each panel of experiment are depicted on the top of the gel with the asterisk indicating the site of the ^32^P label.

The 9-1-1 complex is loaded onto the DNA substrate with the help of the clamp loader RAD17/RFC and we noted that a molar excess of 9-1-1 over DNA2 and EXO1 was required for stimulation of the nucleases. Therefore, we reasoned that the large excess of 9-1-1 required (Figure 1A–D) might be due to rapid binding, sliding and dissociation of 9-1-1 from the linear substrates in the absence of the clamp loader. To test this, we examined the effect of ATP-dependent RAD17/RFC clamp loader on DNA2 cleavage activity. Importantly, RAD17/RFC allowed efficient, ATP-dependent stimulation of DNA2 by 9-1-1, reducing the amount of 9-1-1 required by 5–10-fold (Figure 2A; compare lanes 2 and 5 with lanes 9 and 10). At high concentrations of 9-1-1, as expected, RAD17/RFC had no additive effect on DNA2 cleavage activity (Figure 2A; compare lanes 13 and 16). This suggests that the 9-1-1 complex must be loaded onto the DNA to stimulate DNA2 nuclease activity. To directly measure the interaction of the 9-1-1 complex with the DNA substrate, we performed electromobility gel shift assay in the presence and absence of RAD17/RFC. Low amounts (25 fmol) of 9-1-1 could only bind the flap substrate stably in the presence of RAD17/RFC (Figure 2B; compare lanes 2 and 7). However, 250-fmol 9-1-1 could bind the substrate on its own, in the absence of the clamp loader (Figure 2B, lanes 4 and 5).

To further test whether 9-1-1 needs to be loaded onto DNA for its stimulation of nuclease activity, we examined the effect of blocking the DNA ends. Importantly, we found that blocking the entry of 9-1-1 into the double-stranded region of the substrate by biotin-streptavidin stopped the stimulation of DNA2 by 9-1-1 (Figure 2C; compare lanes 2 and 3 and lanes 4–7). We conclude that 9-1-1 stimulation of nuclease requires loading of the 9-1-1 complex onto DNA, and this 9-1-1 loading is likely dependent on the RAD17/RFC clamp loader complex *in vivo*.

### The 9-1-1 complex promotes Dna2-Sgs1 and Exo1-dependent resection

**Figure 3.**
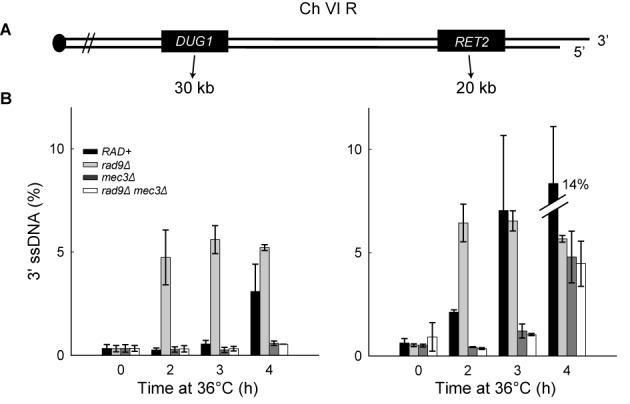
The 9-1-1 complex promotes extensive DNA resection. (**A**) Maps of the right arms of chromosomes VI. (**B**) Analyses of 3′ssDNA accumulation following telomere uncapping in strains lacking Mec3 and/or Rad9 (all in *cdc13-1 cdc15-2 bar1* background). The data and error bars plotted are means and standard deviations from two independent experiments.

We have shown that 9-1-1 increases both DNA2 and EXO1 nuclease activity *in vitro*. These data are to some extent at odds with our previous *in vivo* data, which suggest that 9-1-1 stimulates an unidentified, Exo1-independent nuclease called ExoX (23). Therefore, to clarify whether our new biochemical experiments reflected resection mechanisms active *in vivo*, we used *S. cerevisiae* to re-examine resection near telomeres uncapped by the temperature sensitive allele *cdc13-1.* To examine DNA resection, we measured ssDNA accumulation by QAOS (32) at loci on each right arm telomere of chromosomes VI and V, respectively (Figure 3A and Supplementary Figure S2A). We arrested *cdc13-1* cells harboring various other mutations in G1 with alpha-factor at 23ºC before releasing at 36ºC to induce telomere uncapping. The strains also harbored a *cdc15-2* mutation to keep any checkpoint-deficient strains in late anaphase and ensure a single round of DNA replication during the course of the experiment.

Consistent with previous data (23,26), deletion of *MEC3* (encoding a component of the 9-1-1 complex) reduced ssDNA accumulation following telomere uncapping (Figure 3B and Supplementary Figure S2B; compare *RAD+* and *mec3Δ*), showing that 9-1-1 stimulates resection. We also examined the role of 9-1-1 in a *rad9Δ* background, where resection is increased (26). Consistent with an inhibitory role for Rad9^53BP1^, *rad9Δ* strains started to accumulate 3′ ssDNA at loci distal to the telomeres earlier than a *RAD^+^* strain (Figure 3B and Supplementary Figure S2B). Importantly, in the absence of 9-1-1, deletion of *RAD9* failed to affect ssDNA accumulation (Figure 3B; compare *mec3Δ, rad9Δ* and *rad9Δ mec3Δ* strains). These data suggest that Rad9^53BP1^ inhibits resection at uncapped telomeres entirely by inhibiting 9-1-1-dependent nuclease activities. As expected, ssDNA was specific to the 3′ strands, not 5′ strands (Supplementary Figure S2C), and the quality and quantity of DNA was similar in all samples (Supplementary Figure S2D).

A recent study shows that there is a Cdc13-independent telomere defect in wild-type yeast strains at temperatures above 34ºC (48) raising the possibility that ssDNA generated was not due to *cdc13-*1. Although we have previously reported there is no significant accumulation of ssDNA at telomeres in *CDC^+^* strains at 36°C or 37°C (11,26), we also performed ssDNA measurement experiments at 32ºC. We found that Mec3 and Rad9 played similar roles in resection at 32ºC as at 36°C (Supplementary Figure S3A versus Figure 3B). Together these data show that the 9-1-1 complex supports DNA resection and that Rad9^53BP1^ inhibits resection. As both *rad9Δ* and *mec3Δ* mutants are completely DNA damage checkpoint defective in response to telomere uncapping, as judged the fraction of cells arrested at medial nuclear division after 4 h at 36°C (49), but show different resection phenotypes, the role of the 9-1-1 complex in supporting extensive resection cannot be solely due to checkpoint signaling defects.

**Figure 4.**
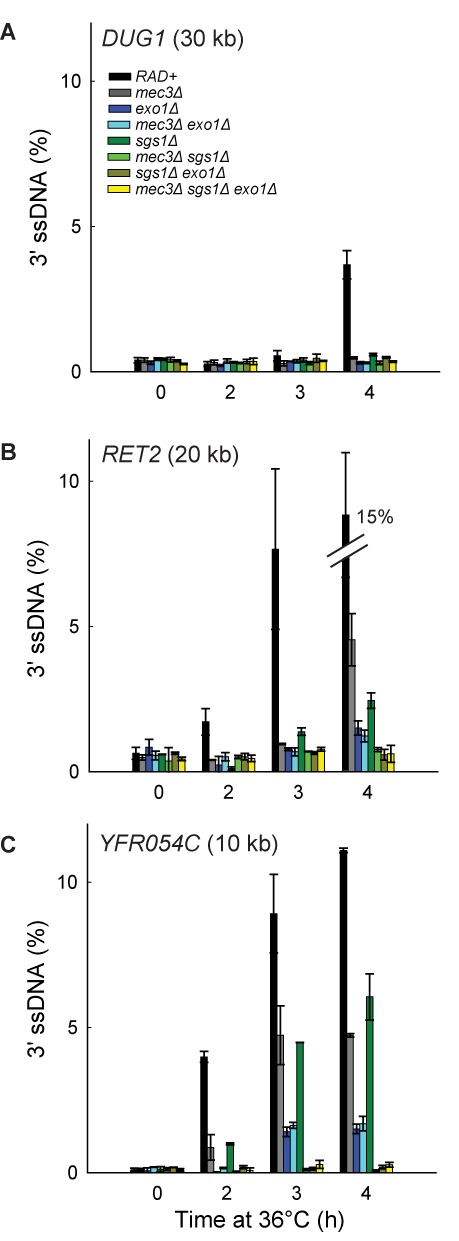
The 9-1-1 complex promotes Exo1-dependent resection. (**A–C**) Analyses of 3′ssDNA accumulation following telomere uncapping in strains lacking Mec3, Sgs1 and/or Exo1 (all in *cdc13-1 cdc15-2 bar1* background).The data and error bars are the means and standard deviations from individual samples measured in triplicate, except *RAD+* and *mec3Δ* strains, where the data and error bars plotted are means and standard deviations from two independent strains.

To begin to determine the interaction between 9-1-1 and Dna2-Sgs1 and/or Exo1-dependent resection *in vivo*, we first examined the roles of Sgs1 and Exo1. We found that deletion of *SGS1* or *EXO1* partially reduced resection at loci distal to the telomeres (Figure 4; compare *RAD+*, *exo1Δ* and *sgs1Δ* strains). Deletion of *EXO1* reduced resection more than deletion of *SGS1*, showing that in this context, Exo1 contributes more to resection than Dna2-Sgs1 (Figure 4B and C). Importantly, deletion of both *SGS1* and *EXO1* completely eliminated resection (Figure 4B and C; compare *RAD+*, *exo1Δ*, *sgs1Δ* and *sgs1Δ exo1Δ* strains). These results show that in *exo1Δ* strains, resection is totally dependent on Dna2-Sgs1 and in *sgs1Δ* strains, resection is totally dependent on Exo1. These results confirm that just like at DSBs (2), extensive telomere resection in *cdc13-1* strains is totally dependent on Dna2-Sgs1 and Exo1 and that it should be informative to examine the effects of 9-1-1 on these nuclease activities.

To test whether 9-1-1 promotes Exo1-dependent resection *in vivo*, we examined how *mec3Δ* affects ssDNA accumulation in *sgs1Δ* strains, where all the resection is due to Exo1. Interestingly, *mec3Δ* completely eliminated ssDNA accumulation in *sgs1Δ* strains (Figure 4B and C; compare *sgs1Δ* with *mec3Δ sgs1Δ* strains). Thus, 9-1-1 appears to be very important for resection by Exo1. This result is consistent with our biochemical data reported above and shows that 9-1-1 can indeed stimulate Exo1 *in vivo*, besides stimulating another Exo1-independent activity (23).

To determine whether 9-1-1 promotes Dna2-Sgs1-dependent resection, we examined how *mec3Δ* affects ssDNA accumulation in *exo1Δ* strains, where all the resection is due to Dna2-Sgs1. We found that *mec3Δ* did not significantly affect resection in *exo1Δ* strains (Figure 4B and C; compare *exo1Δ* with *mec3Δ exo1Δ* strains). Thus, it seems that 9-1-1 is not important for Dna2-Sgs1-dependent resection.

The results so far suggest that 9-1-1 is important for Exo1-dependent but not for Dna2-Sgs1-dependent resection. This is surprising given that our biochemical data suggest that 9-1-1 can stimulate both DNA2 and EXO1 *in vitro* (Figure 1). Furthermore, our previous published data suggest that 9-1-1 stimulated an Exo1-independent activity (23). Therefore, we wondered whether the role of 9-1-1 in Dna2-Sgs1-dependent resection might be obscured by the weaker contribution of Dna2-Sgs1 to resection compared to Exo1 (Figure 4C; compare *sgs1Δ* with *exo1Δ* strains). Therefore, we examined resection in *rad9Δ* background strains, where Dna2-Sgs1-dependent resection is significantly increased and where 9-1-1 has been inferred to stimulate an Exo1-independent activity (9,23).

**Figure 5.**
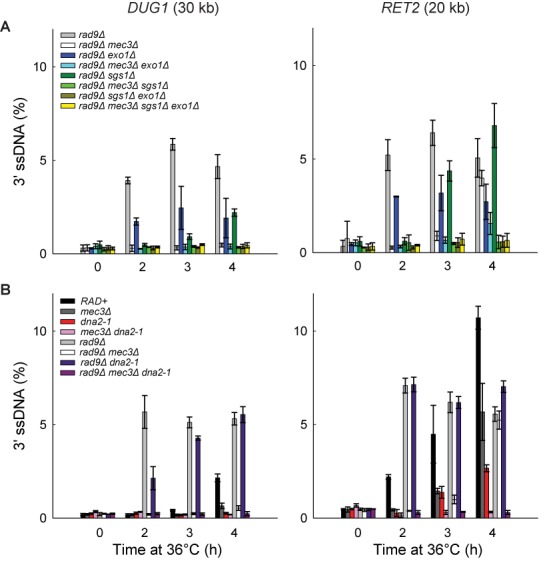
The 9-1-1 complex promotes Exo1- and Dna2-Sgs1-dependent resection. (**A**) Analyses of 3′ssDNA accumulation following telomere uncapping in *rad9Δ* strains lacking Mec3, Sgs1 and/or Exo1 (all in *cdc13-1 cdc15-2 bar1* background). The data and error bars plotted are means and standard deviations from two independent experiments. (**B**) Analyses of 3′ssDNA accumulation following telomere uncapping in strains lacking Mec3, Rad9 and or Dna2 (all in *cdc13-1 cdc15-2 bar1* background). The data and error bars are the means and standard deviations from individual samples measured in triplicate.

As expected and consistent with results in *RAD9+* strains, deletion of *SGS1* or *EXO1* reduced resection in *rad9Δ* strains (Figure 5A). Interestingly, and in contrast to the result in *RAD9+* strains, deletion of *SGS1* reduced resection more than deletion of *EXO1* (Figure 5A; compare *rad9Δ exo1Δ* with *rad9Δ sgs1Δ* strains at 2 h). However, deletion of *EXO1* reduced resection more than deletion of *SGS1* at the late time point (Figure 5A; compare *rad9Δ exo1Δ* with *rad9Δ sgs1Δ* strains at 4 h). This result shows that in the absence of Rad9^53BP1^, Dna2-Sgs1 contributes more to long-range resection than Exo1, whereas in the presence of Rad9^53BP1^, the reverse is true.

Importantly, deletion of both *SGS1* and *EXO1* eliminated resection in *rad9Δ* strains (Figure 5A). These results show that extensive resection observed in *rad9Δ* strains is totally dependent on Dna2-Sgs1 and Exo1, as in *RAD9+* strains. We conclude that deletion of *RAD9* permits Dna2-Sgs1 to be more active than Exo1, but all resection in *rad9Δ* strains is still totally dependent on Dna2-Sgs1 or Exo1.

To determine whether 9-1-1 might promote Dna2-Sgs1-dependent resection in the absence of Rad9^53BP1^, we examined how *mec3Δ* affects ssDNA accumulation in *rad9Δ exo1Δ* strains, where all resection is due to Dna2-Sgs1. Importantly, the *mec3Δ* mutation eliminated resection in *rad9Δ exo1Δ* strains at 2h and 3h time points (Figure 5A; compare *rad9Δ exo1Δ* with *rad9Δ mec3Δ exo1Δ* strains). This result shows that 9-1-1 is, in fact, important for Dna2-Sgs1-dependent resection, but this 9-1-1 effect is only revealed when *RAD9* is deleted.

To test whether 9-1-1 promotes Exo1-dependent resection in the absence of Rad9^53BP1^, we examined how *mec3Δ* affects ssDNA accumulation in *rad9Δ sgs1Δ* strains, where all resection is due to Exo1. Similar to the results obtained in *RAD9+ sgs1Δ* strains, *the mec3Δ* mutation completely eliminated resection in *rad9Δ sgs1Δ* strains (Figure 5A; compare *rad9Δ sgs1Δ* with *rad9Δ mec3Δ sgs1Δ* strains), confirming that 9-1-1 is important for Exo1-dependent resection, whether or not Rad9^53BP1^ is present. Importantly, in support of our hypothesis that Rad9^53BP1^ inhibits resection at uncapped telomeres entirely by inhibiting 9-1-1-dependent nuclease activities (Figure 3), deletion of *RAD9* failed to affect ssDNA accumulation in *mec3Δ exo1Δ* or *mec3Δ sgs1Δ* strains (Figures 4A and B and 5A and Supplementary Figure S3B).

Finally to confirm that 9-1-1 promotes Exo1-dependent resection using a different mutation, we also examined how *mec3Δ* affects ssDNA accumulation in the absence of Dna2 activity, using the *dna2-1* temperature sensitive allele to inactivate Dna2 (because deletion of *DNA2* is lethal). Importantly, the *mec3Δ* mutation completely eliminated ssDNA accumulation in *dna2-1* and *rad9Δ dna2-1* strains (Figure 5B), confirming that 9-1-1 is important for Exo1-dependent resection. Together these data show that 9-1-1 is important for both Dna2-Sgs1 and Exo1-dependent resection. However, Dna2-Sgs1 and Exo1 can also act independently of 9-1-1, as inactivating Sgs1, Dna2 or Exo1 reduced resection in *rad9Δ mec3Δ* strains (Figure 5A and B).

Collectively, all the data in Figures 3–5 show that the 9-1-1 complex stimulates extensive resection by both Dna2-Sgs1 and Exo1 *in vivo* and that Rad9^53BP1^ inhibits this resection stimulatory role of 9-1-1. Furthermore, our results show that ExoX, a 9-1-1 stimulated nuclease, is in fact two nuclease activities, Exo1 and Dna2-Sgs1.

### The 9-1-1 complex promotes the binding of Dna2, Sgs1 and Exo1 to DNA following telomere uncapping

**Figure 6.**
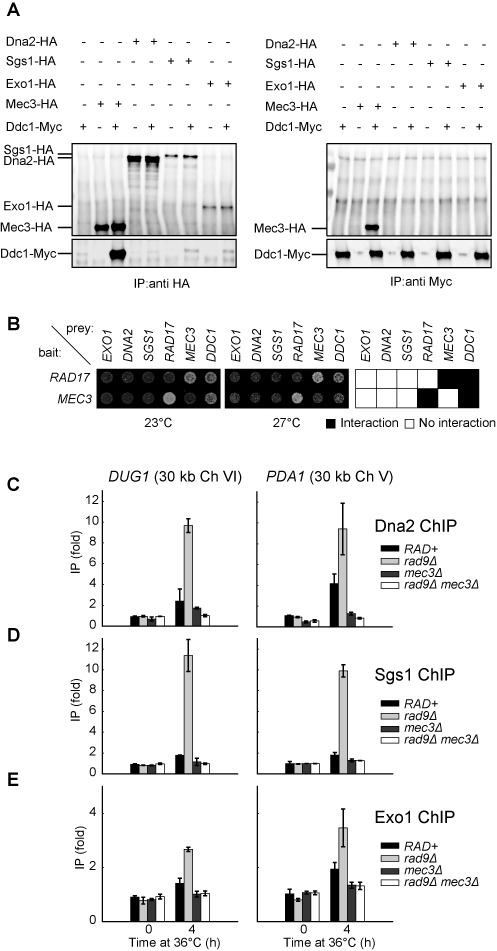
The 9-1-1 complex promotes the binding of Dna2, Sgs1 and Exo1 to DNA following telomere uncapping. (**A**) Co-immunoprecipitation experiment to detect interaction between 9-1-1 and Dna2/Sgs1/Exo1. Protein extract from cells expressing (+) or not expressing (−) the indicated epitope-tagged proteins was subjected to immunoprecipitation with anti-HA (left panel) or anti-Myc (right panel) antibodies, before probing with anti-HA (top panels) or anti-Myc (bottom panels) antibodies. (**B**) Two-hybrid analysis in *cdc13-1* reporter strains at 23°C and 27°C. Bait plasmid contains either *RAD17* or *MEC3* and prey plasmids contain the sequence of the genes indicated. (**C–E**) ChIP analyses of Dna2-Myc, Sgs1-Myc and Exo1-Myc binding to *DUG1* and *PDA1* following telomere uncapping. The data plotted are fold increase over a control locus *PAC2* and represent the means and standard deviations (error bars) from individual samples measured in triplicate.

To test whether 9-1-1 acts directly to stimulate Dna2-Sgs1 and Exo1-dependent resection *in vivo*, we performed co-immunoprecipitation experiments and Y2H assays to detect physical interaction between 9-1-1 and Dna2, Sgs1 or Exo1. We prepared cell extracts from strains expressing Ddc1-Myc, Mec3-HA, Dna2-HA, Sgs1-HA and/or Exo1-HA, and performed immunoprecipitation and detection of these proteins using anti-HA or anti-Myc antibodies. We found that Mec3-HA pulled down Ddc1-Myc and vice versa (Figure 6A) as previously reported (50), suggesting that a Ddc1 or Mec3 pull down brings down the whole 9-1-1 complex. However we detected no interactions between Dna2-HA, Sgs1-HA or Exo1-HA and Ddc1-Myc by IP using anti-HA or anti-Myc antibodies (Figure 6A and Supplementary Figure S4A). Therefore we are unable to detect stable interactions between the 9-1-1 complex and Dna2, Sgs1 or Exo1.

We also considered the possibility that interactions between the 9-1-1 complex and Dna2, Sgs1 or Exo1 might be transient and difficult to detect by co-immunoprecipitation, but might be detectable by Y2H assay. We also introduced *cdc13-1* into the two-hybrid assay to allow us to measure protein interactions in response to temperature regulated telomere uncapping. We detected clear interactions between the components of the 9-1-1 complex, Ddc1, Mec3 and Rad17 (Figure 6B and Supplementary Figure S5). However, we failed to detect any interaction between 9-1-1 components and Dna2, Sgs1 or Exo1 (Figure 6B and Supplementary Figure S5). Interestingly the interactions between Ddc1 and Rad17 or Mec3, but not those between Rad17 and Mec3, seem to be less strong at 27°C than at 23°C, suggesting, perhaps that uncapped telomeres reduce the strength of Ddc1 interactions (Figure 6B and Supplementary Figure S5). We conclude that 9-1-1 does not interact stably with Dna2, Sgs1 or Exo1 in budding yeast.

Our biochemical data suggest that 9-1-1 increases the activity of DNA2 and EXO1 by increasing the local concentration of these proteins. To test whether this is also true *in vivo*, we performed ChIP to examine how 9-1-1 affects the binding of Dna2, Exo1 and Sgs1 to DNA following telomere uncapping. We found that 4 h following telomere uncapping, there was a small increase of Dna2 binding to *DUG1* and *PDA1*, 30 kb from telomeres, in *RAD+* strains (Figure 6C; compare 0 h with 4 h). Interestingly, in *rad9Δ* strains, there was a greater increase in Dna2 binding compared to *RAD+* strains (Figure 6C; compare *rad9Δ* with *RAD+* strains), which may explain the increased ssDNA detected at these loci in *rad9Δ* mutants. Strikingly, *mec3Δ* suppressed this increased Dna2 binding especially in *rad9Δ* mutants (Figure 6C; compare *rad9Δ mec3Δ* with *rad9Δ* strains). These observations suggest that 9-1-1 promotes resection by stimulating Dna2 binding to these loci. We also tested whether the clamp loader for the 9-1-1 complex, Rad24 (yeast ortholog of RAD17) participates in this process. Deletion of *RAD24* reduced Dna2 binding to *DUG1* and *PDA1* (Supplementary Figure S4B), suggesting that 9-1-1 needs to be loaded onto DNA by the Rad24 clamp loader to stimulate Dna2 binding. We also examined the binding of Sgs1 and Exo1 to these loci following telomere uncapping (Figure 6D and E). Importantly, we found that 9-1-1 and Rad9^53BP1^ also affected the binding of both Sgs1 and Exo1 to these loci in a manner similar to Dna2 (Figure 6D and E). These results suggest that 9-1-1 binding to DNA (Supplementary Figure S4C) stimulates the association of Dna2-Sgs1 and Exo1 with DNA. Collectively, our ChIP data support our biochemical and genetic data and suggest that the 9-1-1 complex promotes extensive resection of uncapped telomeres *in vivo* by stimulating the association of Dna2-Sgs1 and Exo1 with DNA.

## DISCUSSION

**Figure 7.**
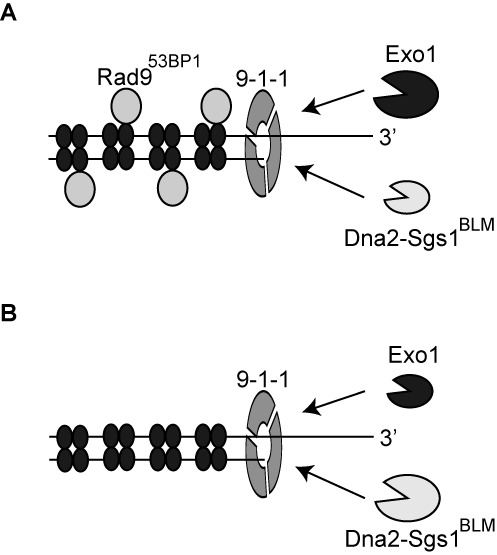
The role of the 9-1-1 complex in stimulating DNA resection. (**A, B**) The 9-1-1 complex stimulates extensive DNA resection by recruiting Dna2-Sgs1^BLM^ and Exo1 to sites of resection. Following recruitment by 9-1-1, Dna2-Sgs1^BLM^ and Exo1 contribute differently to resection. In the presence of Rad9^53BP1^(A), extensive resection is more dependent on Exo1 than Dna2-Sgs1^BLM^. In the absence of Rad9^53BP1^(B), extensive resection is more dependent on Dna2-Sgs1^BLM^ than Exo1. The set of four filled ellipses represent histone octamers.

Here we combine biochemical and genetic analyses to demonstrate for the first time that the 9-1-1 checkpoint clamp complex is an important stimulatory factor for both Dna2-Sgs1 and Exo1 during DNA resection. Our biochemical experiments using purified human proteins and our ChIP experiments in budding yeast show that the 9-1-1 complex performs a resection-stimulatory role by increasing the effective local concentration of Dna2-Sgs1 and Exo1 on DNA (Figure 7A and B). The binding of 9-1-1 to DNA thus generates a positive feedback loop for DNA damage checkpoint activation by promoting resection and ssDNA generation. Other mechanisms, such as Rad53-dependent Exo1 phosphorylation, work in the opposite direction, to inhibit ssDNA accumulation (21).

We propose that the 9-1-1 complex, once loaded onto DNA by the clamp loader, recruits Dna2-Sgs1 or Exo1 to DNA. Although we could detect no interaction between 9-1-1 and these resection proteins, others have shown that Mec3 interacts physically with Exo1 (51). So it is possible that 9-1-1 directly interacts with Exo1 to facilitate its recruitment or prevent its disengagement from DNA. Alternatively, 9-1-1 may alter DNA conformation to facilitate the recruitment/activation of Dna2-Sgs1 or Exo1. This mode of stimulation would be similar to how MRX and SOSS1 stimulate Exo1/EXO1 without direct interactions between the proteins (52,53).

We found that 9-1-1-dependent resection is strongly inhibited by Rad9^53BP1^. This is perhaps because Rad9^53BP1^ binds to chromatin near DNA damage and thereby inhibits Dna2-Sgs1 and Exo1 (22,54). Interestingly, the effect of 9-1-1 in stimulating Dna2-Sgs1-dependent resection is most easily observed in *rad9Δ* background strains. We believe this is because in the presence of Rad9^53BP1^, extensive resection is more dependent on Exo1 than Dna2-Sgs1 (Figure 7A). In contrast, in the absence of Rad9^53BP1^, extensive resection is more dependent on Dna2-Sgs1 than Exo1 (Figure 7B). We propose that, *in vivo*, Exo1 and Dna2-Sgs1 have distinct properties that are most clearly illustrated by the effect of Rad9^53BP1^. If Rad9^53BP1^ is present then Exo1 is the most important nuclease, responsible for most extensive resection, and Dna2-Sgs1 is less important. However, when Rad9^53BP1^ is absent the situation is reversed and Dna2-Sgs1 is responsible for the rapid resection that is observed in *rad9Δ* strains. Since Rad9^53BP1^ binds to chromatin via methylated histone H3K79 and phosphorylated histone H2A, we think that one explanation for this is that the two nucleases have different abilities to resect through Rad9^53BP1^ containing chromatin. We suggest that Exo1 is strong but slow whereas Dna2-Sgs1 is fast but weak. We further suggest that the strong nuclease activity of Exo1 is particularly important for resecting through ‘difficult’ Rad9^53BP1^-dependent chromatin areas, whereas Dna2-Sgs1 can rapidly move through less difficult areas. We propose that Rad9^53BP1^ may bind with different affinity to different regions of the genome due to chromatin differences. If so, and if resection is to be extensive and efficient, it will depend on both Exo1 and Dna2-Sgs1 nuclease activities. According to this model, 9-1-1 may play a critical role in the changeover between nuclease activities, helping to recruit nuclease(s) with different properties at the ss/dsDNA junction, just as PCNA, the replicative sliding clamp that recruits different activities to the replication fork (55).

53BP1, the mammalian homolog of Rad9, with its interacting proteins (RIF1 and PTIP) also inhibits DNA resection, but the exact mechanism of inhibition remains unclear (56–61). It will be interesting to test whether 9-1-1 promotes resection by DNA2-BLM and EXO1 in mammalian cells and if so whether 9-1-1 is responsible for increased resection in cells lacking 53BP1.

In conclusion, we provide novel mechanistic insights into how the important function of DNA resection is regulated by the 9-1-1 complex. Our results have important implications for the mechanisms that maintain genome stability and potentially shed light on the involvement of the 9-1-1 complex in many other processes like telomere maintenance, repair of stalled replication forks, homologous recombination and cancer progression (51,62–67).

## SUPPLEMENTARY DATA

Supplementary Data are available at NAR Online.

SUPPLEMENTARY DATA
