# Supplementary material for: The 9-1-1 checkpoint clamp stimulates DNA resection by Dna2-Sgs1 and Exo1
Source: Nucleic Acids Res. 2014 Aug 13;42(16):10516–28. doi: 10.1093/nar/gku746 (PMC4176354; doi:10.1093/nar/gku746)
Supplement: SUPPLEMENTARY DATA [file supp_42_16_10516__index.html]

The 9-1-1 checkpoint clamp stimulates DNA resection by Dna2-Sgs1 and Exo1 — SUPPLEMENTARY DATA 

# The 9-1-1 checkpoint clamp stimulates DNA resection by Dna2-Sgs1 and Exo1

## SUPPLEMENTARY DATA

**Files in this Data Supplement:**

- SUPPLEMENTARY DATA
