# Supplementary material for: The 9-1-1 checkpoint clamp stimulates DNA resection by Dna2-Sgs1 and Exo1
Source: Nucleic Acids Res. 2014 Aug 13;42(16):10516–28. doi: 10.1093/nar/gku746 (PMC4176354; doi:10.1093/nar/gku746)
Supplement: SUPPLEMENTARY DATA [file supp_gku746_nar-01645-d-2014-File009.pdf]

Figure S1

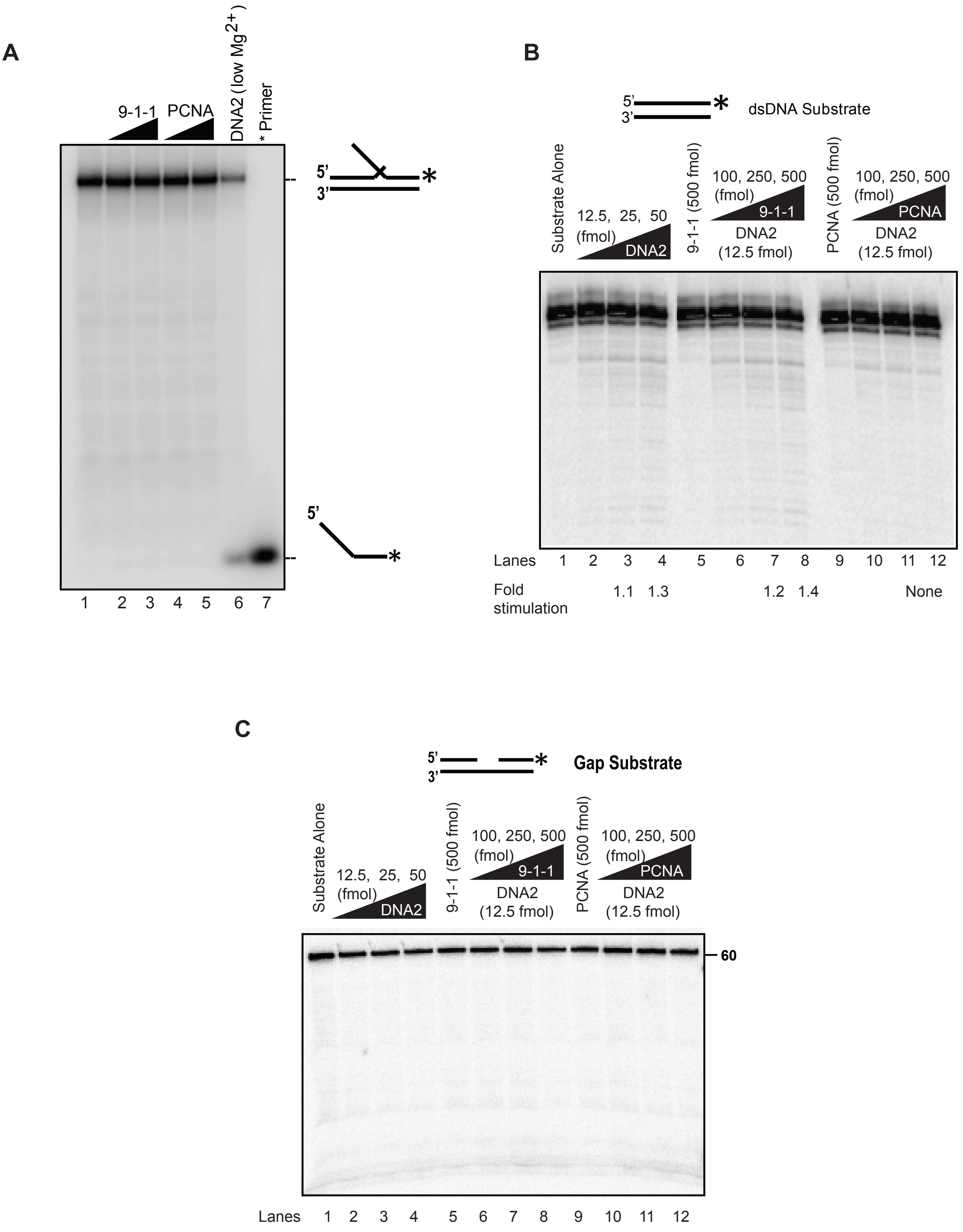

A
9-1-1
PCNA
DNA2 (low Mg^{2+})
* Primer
5'
3'
*
1 2 3 4 5 6 7
B
5'
3'
dsDNA Substrate
Substrate Alone
12.5, 25, 50 (fmol)
DNA2
9-1-1 (500 fmol)
100, 250, 500 (fmol)
9-1-1
DNA2 (12.5 fmol)
PCNA (500 fmol)
100, 250, 500 (fmol)
PCNA
DNA2 (12.5 fmol)
Lanes 1 2 3 4 5 6 7 8 9 10 11 12
Fold stimulation 1.1 1.3 1.2 1.4 None
C
5'
3'
Gap Substrate
Substrate Alone
12.5, 25, 50 (fmol)
DNA2
9-1-1 (500 fmol)
100, 250, 500 (fmol)
9-1-1
DNA2 (12.5 fmol)
PCNA (500 fmol)
100, 250, 500 (fmol)
PCNA
DNA2 (12.5 fmol)
60
Lanes 1 2 3 4 5 6 7 8 9 10 11 12

Figure S2

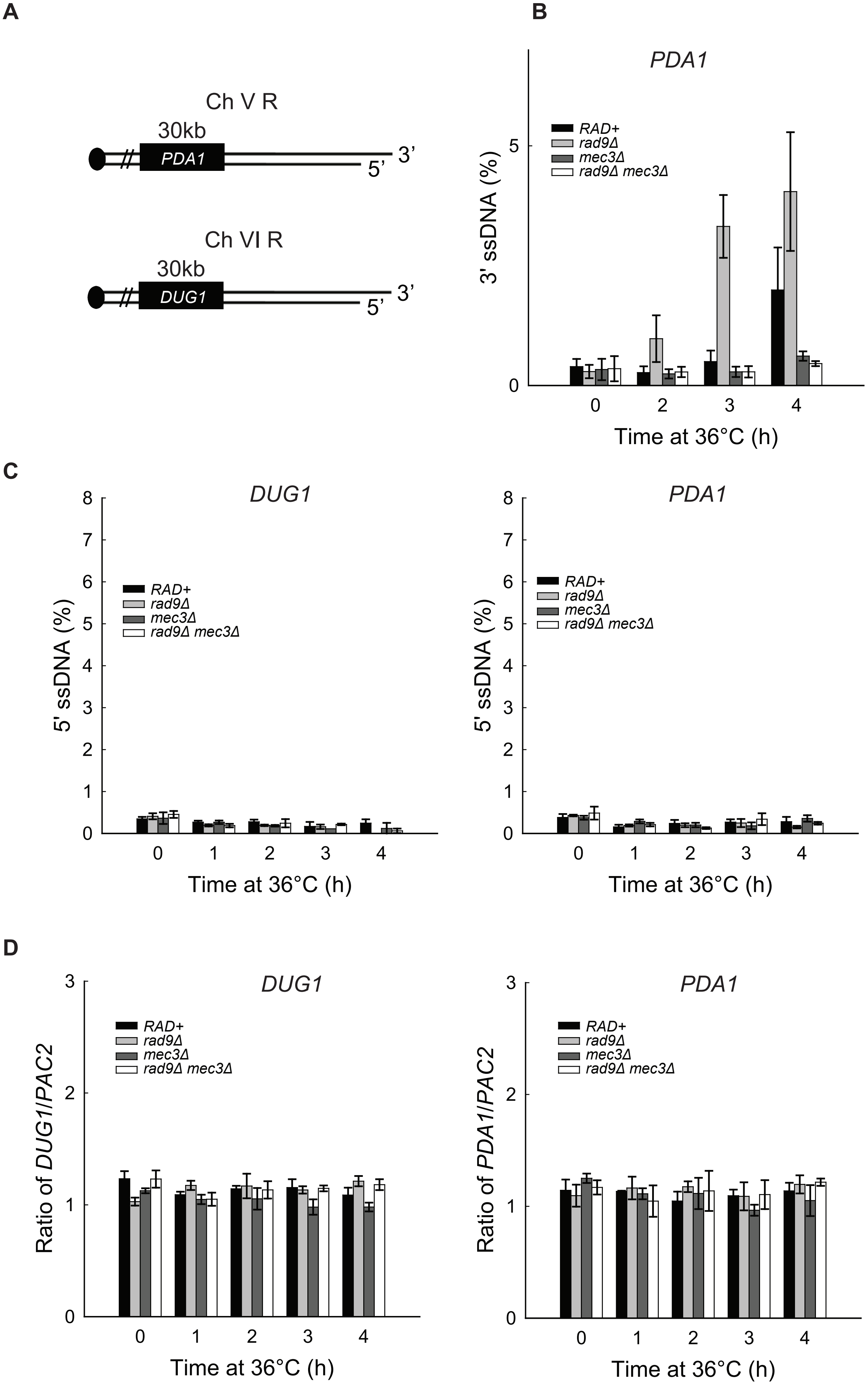
A
Ch V R
30kb
PDA1
3'
5'
Ch VI R
30kb
DUG1
3'
5'
B
PDA1
RAD+
rad9Δ
mec3Δ
rad9Δ mec3Δ
3' ssDNA (%)
Time at 36°C (h)
C
DUG1
PDA1
5' ssDNA (%)
Time at 36°C (h)
D
DUG1
PDA1
Ratio of DUG1/PAC2
Ratio of PDA1/PAC2
Time at 36°C (h)

Figure S3

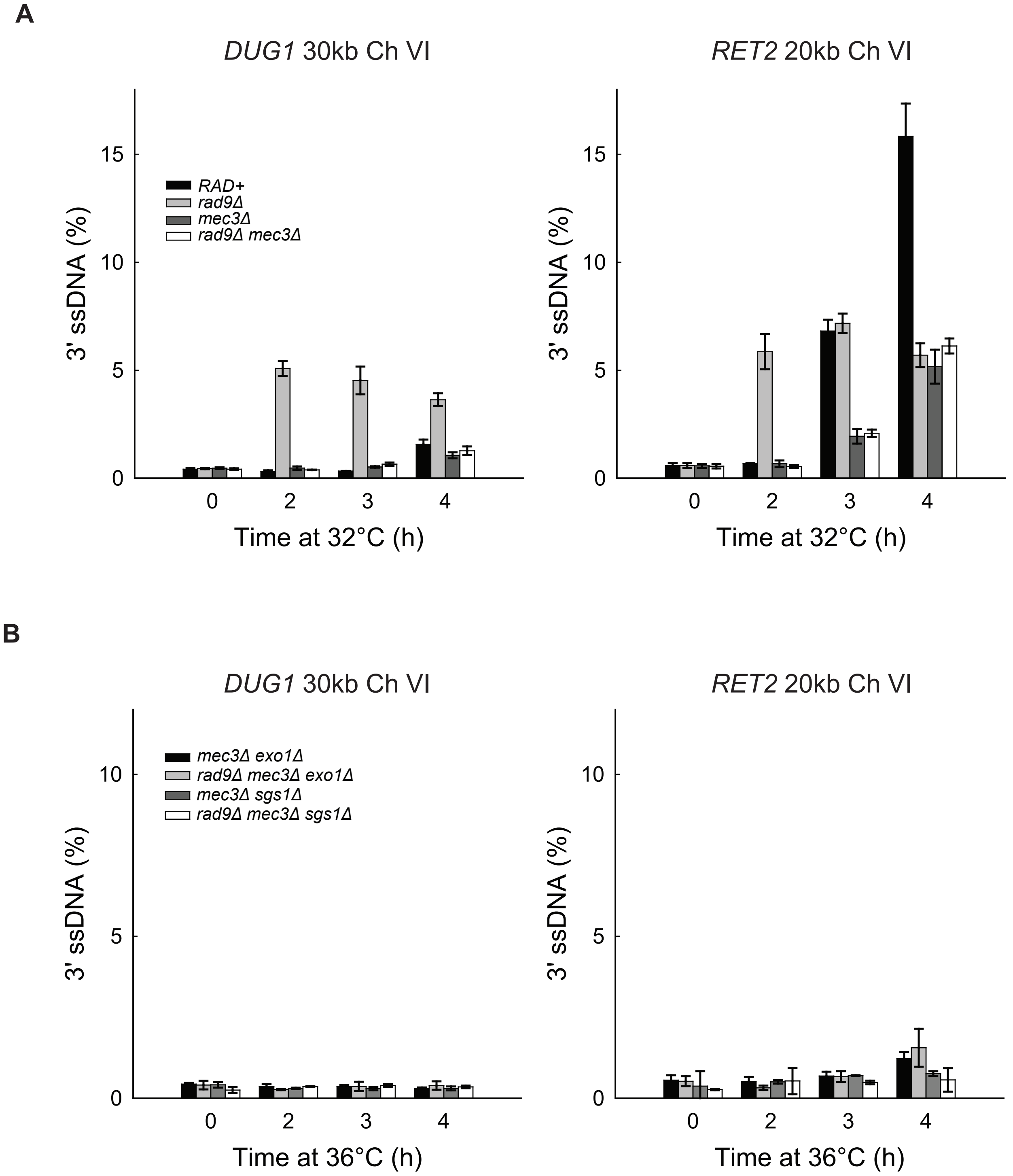
A
DUG1 30kb Ch VI
RET2 20kb Ch VI
RAD+
rad9Δ
mec3Δ
rad9Δ mec3Δ
3' ssDNA (%)
0
5
10
15
0
2
3
4
Time at 32°C (h)
B
DUG1 30kb Ch VI
RET2 20kb Ch VI
mec3Δ exo1Δ
rad9Δ mec3Δ exo1Δ
mec3Δ sgs1Δ
rad9Δ mec3Δ sgs1Δ
3' ssDNA (%)
0
5
10
0
2
3
4
Time at 36°C (h)

Figure S4

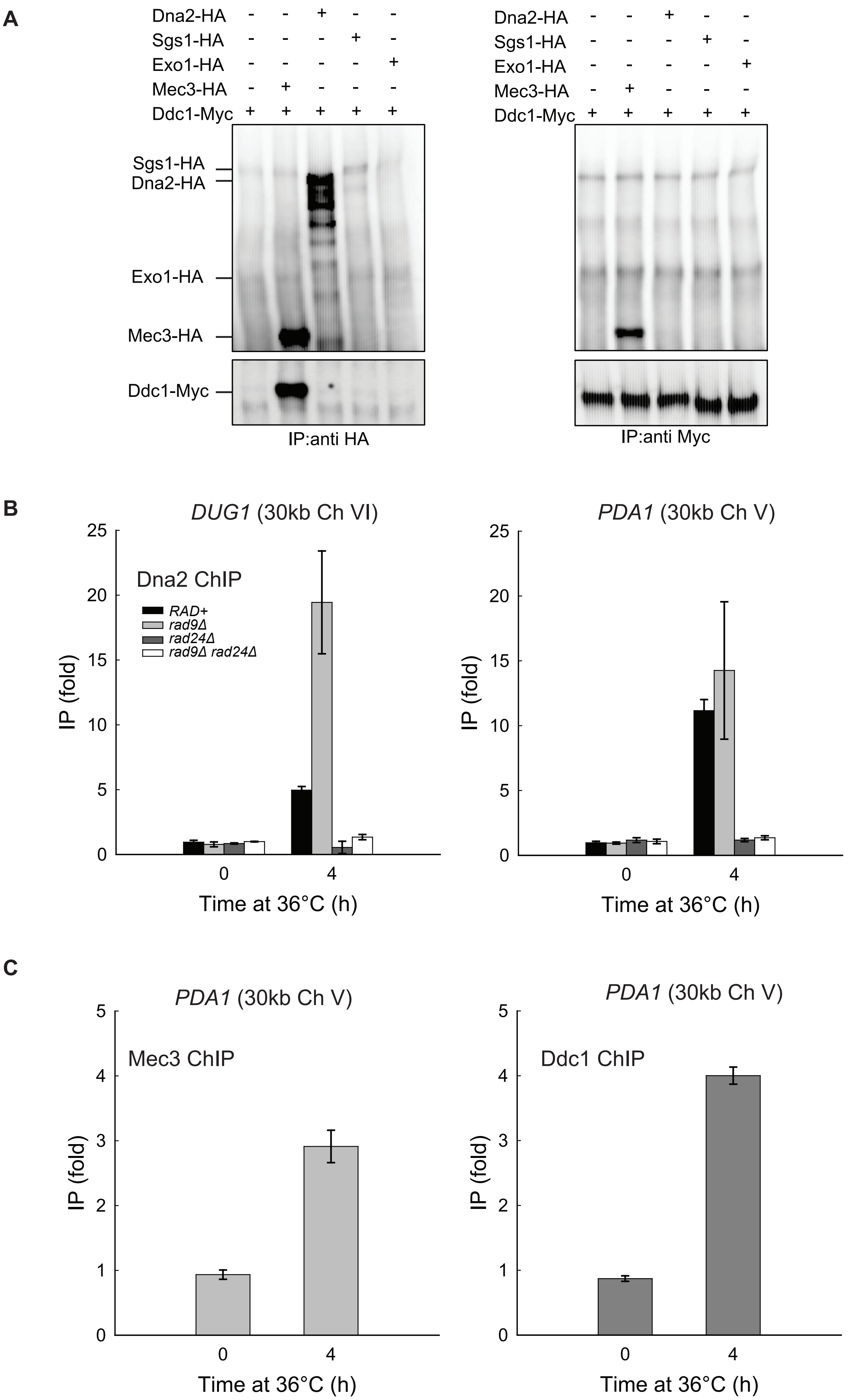
A
Dna2-HA - - + - -
Sgs1-HA - - - + -
Exo1-HA - - - - +
Mec3-HA - + - - -
Ddc1-Myc + + + + +
Sgs1-HA
Dna2-HA
Exo1-HA
Mec3-HA
Ddc1-Myc
IP:anti HA
IP:anti Myc
B
DUG1 (30kb Ch VI)
PDA1 (30kb Ch V)
Dna2 ChIP
RAD+
rad9Δ
rad24Δ
rad9Δ rad24Δ
IP (fold)
Time at 36°C (h)
0
4
5
10
15
20
25
C
PDA1 (30kb Ch V)
Mec3 ChIP
Ddc1 ChIP
IP (fold)
Time at 36°C (h)
1
2
3
4
5

Figure S5

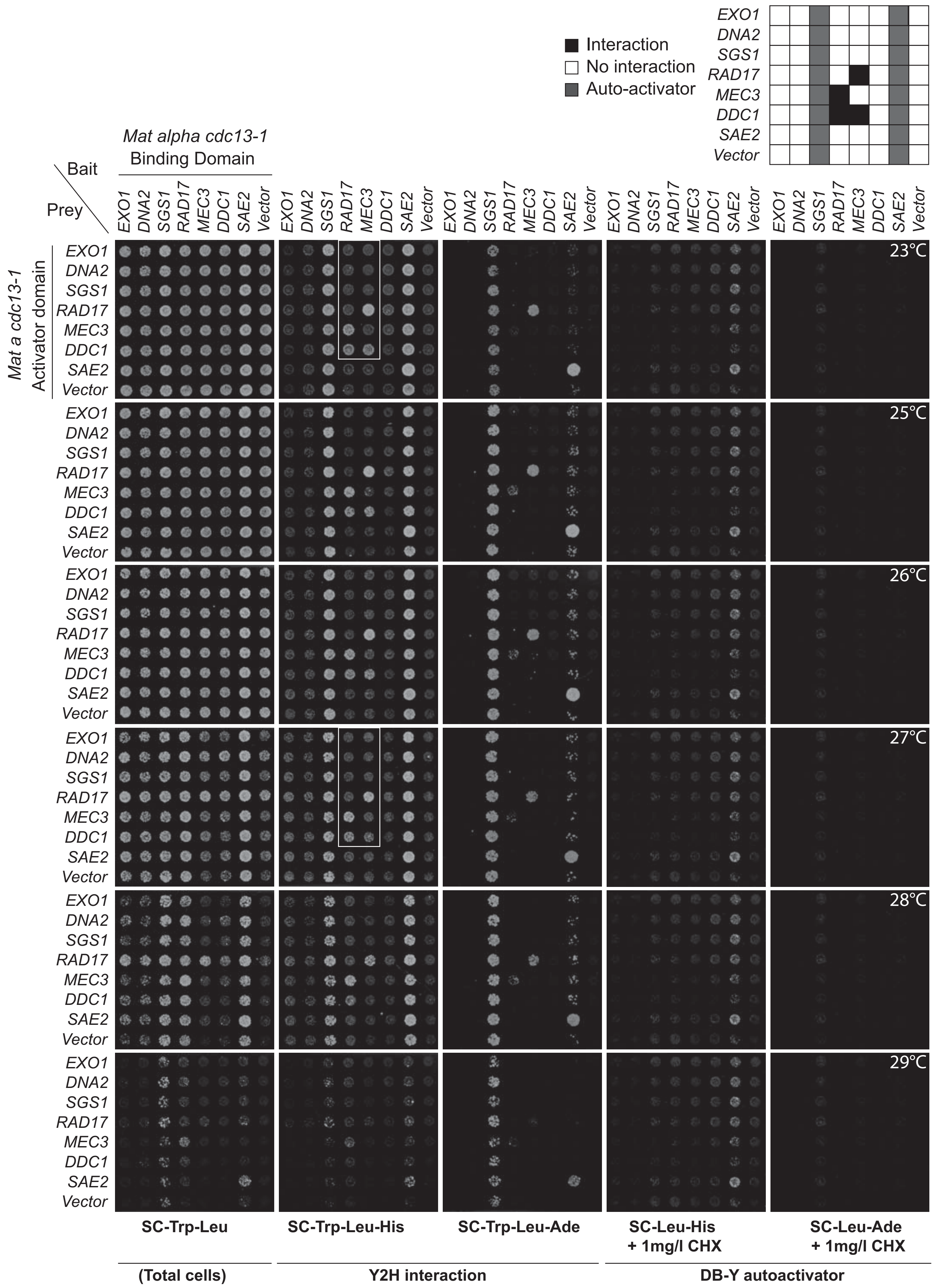
Interaction
No interaction
Auto-activator
EXO1
DNA2
SGS1
RAD17
MEC3
DDC1
SAE2
Vector
Mat alpha cdc13-1
Binding Domain
Bait
Prey
Mat a cdc13-1
Activator domain
23°C
25°C
26°C
27°C
28°C
29°C
SC-Trp-Leu
SC-Trp-Leu-His
SC-Trp-Leu-Ade
SC-Leu-His
+ 1mg/l CHX
SC-Leu-Ade
+ 1mg/l CHX
(Total cells)
Y2H interaction
DB-Y autoactivator

Table S1

Strains	Genotype	Source
DLY7830	*MAT a ade2-1 trp1-1 can1-100 leu2-3,112 his3-11,15 ura3* GAL+ psi+ ssd1-d2 RAD5 *cdc13-1 cdc15-2 bar1*	This work, Fig.3
DLY7834	*MAT a ade2-1 trp1-1 can1-100 leu2-3,112 his3-11,15 ura3* GAL+ psi+ ssd1-d2 RAD5 *cdc13-1 cdc15-2 bar1 rad9Δ:::HIS3*	This work, Fig.3
DLY7960	*MAT a ade2-1 trp1-1 can1-100 leu2-3,112 his3-11,15 ura3* GAL+ psi+ ssd1-d2 RAD5 *cdc13-1 cdc15-2 bar1 mec3Δ:::TRP1*	This work, Fig.3
DLY7964	*MAT a ade2-1 trp1-1 can1-100 leu2-3,112 his3-11,15 ura3* GAL+ psi+ ssd1-d2 RAD5 *cdc13-1 cdc15-2 bar1 rad9Δ:::HIS3 mec3Δ:::TRP1*	This work, Fig.3
DLY7115	*MAT a ade2-1 trp1-1 can1-100 leu2-3,112 his3-11,15 ura3* GAL+ psi+ ssd1-d2 RAD5 *cdc13-1 cdc15-2 bar1 dna2-1*	This work, Fig.5
DLY7813	*MAT a ade2-1 trp1-1 can1-100 leu2-3,112 his3-11,15 ura3* GAL+ psi+ ssd1-d2 RAD5 *cdc13-1 cdc15-2 bar1 rad9Δ:::HIS3 dna2-1*	This work, Fig.5
DLY7967	*MAT a ade2-1 trp1-1 can1-100 leu2-3,112 his3-11,15 ura3* GAL+ psi+ ssd1-d2 RAD5 *cdc13-1 cdc15-2 bar1 mec3Δ:::TRP1 dna2-1*	This work, Fig.5
DLY7973	*MAT a ade2-1 trp1-1 can1-100 leu2-3,112 his3-11,15 ura3* GAL+ psi+ ssd1-d2 RAD5 *cdc13-1 cdc15-2 bar1 rad9Δ:::HIS3 mec3Δ:::TRP1 dna2-1*	This work, Fig.5
DLY7837	*MAT a ade2-1 trp1-1 can1-100 leu2-3,112 his3-11,15 ura3* GAL+ psi+ ssd1-d2 RAD5 *cdc13-1 cdc15-2 bar1 rad9::HIS3 exo1Δ:::LEU2*	This work, Fig.5
DLY8080	*MAT a ade2-1 trp1-1 can1-100 leu2-3,112 his3-11,15 ura3* GAL+ psi+ ssd1-d2 RAD5 *cdc13-1 cdc15-2 bar1 rad9::HIS3 exo1Δ::LEU2 mec3Δ:::TRP1*	This work, Fig.5
DLY5267	*MAT a ade2-1 trp1-1 can1-100 leu2-3,112 his3-11,15 ura3* GAL+ psi+ ssd1-d2 RAD5 *cdc13-1 cdc15-2 bar1 rad9::HIS3 sgs1Δ:::KanMX*	This work, Fig.5
DLY8547	*MAT a ade2-1 trp1-1 can1-100 leu2-3,112 his3-11,15 ura3* GAL+ psi+ ssd1-d2 RAD5 *cdc13-1 cdc15-2 bar1 rad9::HIS3 mec3::TRP1 sgs1Δ:::KanMX*	This work, Fig.5
DLY5273	*MAT a ade2-1 trp1-1 can1-100 leu2-3,112 his3-11,15 ura3* GAL+ psi+ ssd1-d2 RAD5 *cdc13-1 cdc15-2 bar1 rad9::HIS3 sgs1Δ:::KanMX exo1Δ:::LEU2*	This work, Fig.5
DLY8615	*MAT a ade2-1 trp1-1 can1-100 leu2-3,112 his3-11,15 ura3* GAL+ psi+ ssd1-d2 RAD5 *cdc13-1 cdc15-2 bar1 rad9::HIS3 sgs1Δ:::KanMX exo1Δ:::LEU2 mec3::TRP1*	This work, Fig.5
DLY8019	*MAT a ade2-1 trp1-1 can1-100 leu2-3,112 his3-11,15 ura3* GAL+ psi+ ssd1-d2 RAD5 *cdc13-1 cdc15-2 bar1 Dna2-Myc-KanMX*	This work, Fig.6
DLY8022	*MAT a ade2-1 trp1-1 can1-100 leu2-3,112 his3-11,15 ura3* GAL+ psi+ ssd1-d2 RAD5 *cdc13-1 cdc15-2 bar1 Dna2-Myc-KanMX rad9Δ:::LEU2*	This work, Fig.6
DLY8024	*MAT a ade2-1 trp1-1 can1-100 leu2-3,112 his3-11,15 ura3* GAL+ psi+ ssd1-d2 RAD5 *cdc13-1 cdc15-2 bar1 Dna2-Myc-KanMX mec3Δ:::TRP1*	This work, Fig.6
DLY8027	*MAT a ade2-1 trp1-1 can1-100 leu2-3,112 his3-11,15 ura3* GAL+ psi+ ssd1-d2 RAD5 *cdc13-1 cdc15-2 bar1 Dna2-Myc-KanMX rad9Δ:::LEU2 mec3Δ:::TRP1*	This work, Fig.6
DLY8587	*MAT a ade2-1 trp1-1 can1-100 leu2-3,112 his3-11,15 ura3* GAL+ psi+ ssd1-d2 RAD5 *cdc13-1 cdc15-2 bar1 Sgs1-Myc-HphNT1*	This work, Fig.6
DLY8589	*MAT a ade2-1 trp1-1 can1-100 leu2-3,112 his3-11,15 ura3* GAL+ psi+ ssd1-d2 RAD5 *cdc13-1 cdc15-2 bar1 Sgs1-Myc-HphNT1 rad9Δ:::LEU2*	This work, Fig.6
DLY8591	*MAT a ade2-1 trp1-1 can1-100 leu2-3,112 his3-11,15 ura3* GAL+ psi+ ssd1-d2 RAD5 *cdc13-1 cdc15-2 bar1 Sgs1-Myc-HphNT1 mec3Δ:::TRP1*	This work, Fig.6
DLY8593	*MAT a ade2-1 trp1-1 can1-100 leu2-3,112 his3-11,15 ura3* GAL+ psi+ ssd1-d2 RAD5 *cdc13-1 cdc15-2 bar1 Sgs1-Myc-HphNT1 rad9Δ:::LEU2 mec3Δ:::TRP1*	This work, Fig.6
DLY8033	*MAT a ade2-1 trp1-1 can1-100 leu2-3,112 his3-11,15 ura3* GAL+ psi+ ssd1-d2 RAD5 *cdc13-1 cdc15-2 bar1 Exo1-Myc-HIS3*	This work, Fig.6
DLY8036	*MAT a ade2-1 trp1-1 can1-100 leu2-3,112 his3-11,15 ura3* GAL+ psi+ ssd1-d2 RAD5 *cdc13-1 cdc15-2 bar1 Exo1-Myc-HIS3 rad9Δ:::LEU2*	This work, Fig.6
DLY8039	*MAT a ade2-1 trp1-1 can1-100 leu2-3,112 his3-11,15 ura3* GAL+ psi+ ssd1-d2 RAD5 *cdc13-1 cdc15-2 bar1 Exo1-Myc- HIS3 mec3Δ:::TRP1*	This work, Fig.6
DLY8042	*MAT a ade2-1 trp1-1 can1-100 leu2-3,112 his3-11,15 ura3* GAL+ psi+ ssd1-d2 RAD5 *cdc13-1 cdc15-2 bar1 Exo1-Myc-HIS3 rad9Δ:::LEU2 mec3Δ:::TRP1*	This work, Fig.6
DLY8045	*MAT a ade2-1 trp1-1 can1-100 leu2-3,112 his3-11,15 ura3* GAL+ psi+ ssd1-d2 RAD5 *cdc13-1 cdc15-2 bar1 Dna2-Myc-KanMX rad24Δ:::TRP1*	This work, Fig.S4
DLY8048	*MAT a ade2-1 trp1-1 can1-100 leu2-3,112 his3-11,15 ura3* GAL+ psi+ ssd1-d2 RAD5 *cdc13-1 cdc15-2 bar1 Dna2-Myc-KanMX rad9::HIS3 rad24::TRP1*	This work, Fig.S4

Table S2

Strains	Genotype	Source
DLY7833	*MAT a ade2-1 trp1-1 can1-100 leu2-3,112 his3-11,15 ura3* GAL+ psi+ ssd1-d2 RAD5 *cdc13-1 cdc15-2 bar1 exo1Δ:::LEU2*	This work, Fig.4
DLY8478	*MAT a ade2-1 trp1-1 can1-100 leu2-3,112 his3-11,15 ura3* GAL+ psi+ ssd1-d2 RAD5 *cdc13-1 cdc15-2 bar1 exo1Δ:::LEU2 mec3Δ:::TRP1*	This work, Fig.4
DLY5123	*MAT a ade2-1 trp1-1 can1-100 leu2-3,112 his3-11,15 ura3* GAL+ psi+ ssd1-d2 RAD5 *cdc13-1 cdc15-2 bar1 sgs1Δ:::KanMX*	This work, Fig.4
DLY8544	*MAT a ade2-1 trp1-1 can1-100 leu2-3,112 his3-11,15 ura3* GAL+ psi+ ssd1-d2 RAD5 *cdc13-1 cdc15-2 bar1 sgs1Δ:::KanMX mec3Δ:::TRP1*	This work, Fig.4
DLY5269	*MAT a ade2-1 trp1-1 can1-100 leu2-3,112 his3-11,15 ura3* GAL+ psi+ ssd1-d2 RAD5 *cdc13-1 cdc15-2 bar1 sgs1Δ:::KanMX exo1Δ:::LEU2*	This work, Fig.4
DLY8548	*MAT a ade2-1 trp1-1 can1-100 leu2-3,112 his3-11,15 ura3* GAL+ psi+ ssd1-d2 RAD5 *cdc13-1 cdc15-2 bar1 sgs1Δ:::KanMX exo1Δ:::LEU2 mec3::TRP1*	This work, Fig.4
DLY7870	*MAT a ade2-1 trp1-1 can1-100 leu2-3,112 his3-11,15 ura3* GAL+ psi+ ssd1-d2 RAD5 *cdc13-1 cdc15-2 bar1 Ddc1-Myc-HIS3MX6*	This work, Fig.4
DLY8971	*MAT a ade2-1 trp1-1 can1-100 leu2-3,112 his3-11,15 ura3* GAL+ psi+ ssd1-d2 RAD5 *cdc13-1 cdc15-2 bar1 Mec3-3HA-KanMX*	This work, Fig.6
DLY8989	*MAT a ade2-1 trp1-1 can1-100 leu2-3,112 his3-11,15 ura3* GAL+ psi+ ssd1-d2 RAD5 *cdc13-1 cdc15-2 bar1 Mec3-3HA-KanMX Ddc1-Myc-HIS3MX6*	This work, Fig.6
DLY7929	*MAT a ade2-1 trp1-1 can1-100 leu2-3,112 his3-11,15 ura3* GAL+ psi+ ssd1-d2 RAD5 *cdc13-1 cdc15-2 bar1 Ddc1-Myc-his3MX6 Dna2-3HA-KanMX*	This work, Fig.6
DLY8983	*MAT a ade2-1 trp1-1 can1-100 leu2-3,112 his3-11,15 ura3* GAL+ psi+ ssd1-d2 RAD5 *cdc13-1 cdc15-2 bar1 Ddc1-Myc-HIS3MX6 Sgs1-3HA-KanMX*	This work, Fig.6
DLY8985	*MAT a ade2-1 trp1-1 can1-100 leu2-3,112 his3-11,15 ura3* GAL+ psi+ ssd1-d2 RAD5 *cdc13-1 cdc15-2 bar1 Ddc1-Myc-HIS3MX6 Exo1-3HA-KanMX*	This work, Fig.6
DLY8963	*MAT a ade2-1 trp1-1 can1-100 leu2-3,112 his3-11,15 ura3* GAL+ psi+ ssd1-d2 RAD5 *cdc13-1 cdc15-2 bar1 Sgs1-3HA-KanMX*	This work, Fig.6
DLY8965	*MAT a ade2-1 trp1-1 can1-100 leu2-3,112 his3-11,15 ura3* GAL+ psi+ ssd1-d2 RAD5 *cdc13-1 cdc15-2 bar1 Dna2-3HA-KanMX*	This work, Fig.6
DLY8967	*MAT a ade2-1 trp1-1 can1-100 leu2-3,112 his3-11,15 ura3* GAL+ psi+ ssd1-d2 RAD5 *cdc13-1 cdc15-2 bar1 Exo1-3HA-KanMX*	This work, Fig.6
Y8800	*Mata leu2-3,112 trp1-901 his3-200 ura3-52 gal4Δ gal80Δ GAL2-ADE2 LYS2::GAL1-HIS3 MET2::GAL7-LacZ cyh2R*	Dreze et.al, Fig.6
Y8930	*Matalpha leu2-3,112 trp1-901 his3-200 ura3-52 gal4Δ gal80Δ GAL2-ADE2 LYS2::GAL1-HIS3 MET2::GAL7-LacZ cyh2R*	Dreze et.al, Fig.6
DLY7451	*Mata leu2-3,112 trp1-901 his3-200 ura3-52 gal4Δ gal80Δ GAL2-ADE2 LYS2::GAL1-HIS3 MET2::GAL7-LacZ cyh2R cdc13-1*	This work, Fig.6
DLY7452	*Matalpha leu2-3,112 trp1-901 his3-200 ura3-52 gal4Δ gal80Δ GAL2-ADE2 LYS2::GAL1-HIS3 MET2::GAL7-LacZ cyh2R cdc13-1*	This work, Fig.6
DLY7909	*MAT a ade2-1 trp1-1 can1-100 leu2-3,112 his3-11,15 ura3* GAL+ psi+ ssd1-d2 RAD5 *cdc13-1 cdc15-2 bar1 Mec3-Myc-HIS3MX6*	This work, Fig.S4
DLY7925	*MAT a ade2-1 trp1-1 can1-100 leu2-3,112 his3-11,15 ura3* GAL+ psi+ ssd1-d2 RAD5 *cdc13-1 cdc15-2 bar1 Ddc1-Myc-HIS3MX6*	This work, Fig.S4

Table S3

Primer	Length (nts)	Sequence
**<u>Upstream</u>**		**<u>Listed 5' to 3'</u>**
U1	26	CGCCAGGGTTTTCCCAGTCACGACCA
**<u>Downstream</u>**		**<u>Listed 5' to 3'</u>**
D1	53	TTCACGCCTGTTAGTTAATTCACTGGCCGTCGTTTTACAACGACGTGACTGG G
D2	55	CGACCGTGCCAGCCTAAAACACTTGCCCGTGCCACCATCCCGACGCCACCT CCTG
D3	60	AGACGAATTCGGATACGACGGCAGTGCCGACCGTCCAGCCTAAATTCAATC CACCC
**<u>Template</u>**		**<u>Listed 3' to 5'</u>**
T1	49	GCGGTCCCAAAAGGGTCAGTGCTGGGCAAAATGTTGCTGCACTGACCCG
T2	56	GCTGGCACGGTCGGATTTTGTGAACGGGCACGGTGGTAGGGCTGCGGTGG AGGACG
T3	110	CAGGTGGGCGCGGTGGAGGACGAAGTTACACGACCTAGGATGTTGTTCTG CTTAAGCCTATGCTCCGGTCACGGCTGCACGGTCGGATTAAAGTTAGGTGG

Table S4

Primer	Sequence 5’-3’	Description
M2588	aagataccccaccaaacccaaaaaaagagggtgggGGTATCCAAGGTCTTCTTCC	*AD-EXO1*
M2589	aaggtcaaagacagttgactgtatcgtcgaggtcg GGTATCCAAGGTCTTCTTCC	*DB-EXO1*
M2590	cttacttagagctcgacgtcttacttacttagcggccgc TTTACCTTTATAAACAAATTGGG	R- *EXO1*
M3100	aagataccccaccaaacccaaaaaaagagggtggg CCCGGAACGCCACAGAAG	*AD-DNA2*
M3099	aaggtcaaagacagttgactgtatcgtcgaggtcgCCCGGAACGCCACAGAAG	*DB-DNA2*
M3101	cttacttagagctcgacgtcttacttacttagcggccgcACTTTCATACTCTTGTAGAA	R-DNA2
M3104	aagataccccaccaaacccaaaaaaagagggtgggGTGACGAAGCCGTCACATAACTTA	*AD-SGS1*
M3103	aaggtcaaagacagttgactgtatcgtcgaggtcgGTGACGAAGCCGTCACATAACTTA	*DB-SGS1*
M2626	TTAAAACTTTGTTTTAGGAAAACAG	*DB-Rev-SGS1*
M2625	CCTTGGTCTGATGAAGTTTTATAT	*DB-Fwd-SGS1*
M2596	cttacttagagctcgacgtcttacttacttagcggccgcTTAAAACTTTGTTTTAGGAAAACAG	R-*SGS1*
M2600	aagataccccaccaaacccaaaaaaagagggtgggTCATTTAAGGCAACTATCACC	*AD-DDC1*
M2602	cttacttagagctcgacgtcttacttacttagcggccgcGTCAAATATACCCCTTGGC	R-*DDC1*
M2603	aagataccccaccaaacccaaaaaaagagggtgggCGAATCAACAGTGAGCTAG	*AD-RAD17*
M2604	aaggtcaaagacagttgactgtatcgtcgaggtcgCGAATCAACAGTGAGCTAG	*DB-RAD17*
M2605	cttacttagagctcgacgtcttacttacttagcggccgcAAAAAATATAGGAATATCCTTTGTTG	R-*RAD17*
M2606	aagataccccaccaaacccaaaaaaagagggtgggAAATTAAAATTGATAGTAAATGGTTGT	*AD-MEC3*
M2607	aaggtcaaagacagttgactgtatcgtcgaggtcgAAATTAAAATTGATAGTAAATGGTTGT	*DB-MEC3*
M2608	cttacttagagctcgacgtcttacttacttagcggccgcCAAGCCCTTCGATCTT	R- *MEC3*
M3106	aagataccccaccaaacccaaaaaaagagggtgggGTGACTGGTGAAGAAAATGTGT	*AD-SAE2*
M3105	aaggtcaaagacagttgactgtatcgtcgaggtcgGTGACTGGTGAAGAAAATGTGT	*DB-SAE2*
M3107	C cttacttagagctcgacgtcttacttacttagcggccgcACATCTAGCATATATCTGCAATAA	R-SAE2

Forward primers generate the fusion of the Gal4 activation domain (*AD-X*) or the Gal4 DNA binding domain (*DB-Y*) with the protein of interest. Each reverse primer (R) can be used with either the AD-X or the DB-Y primers. *DB-SGS1* was cloned using two PCR fragments: M3103-M2626 and M2625-M2596.

SUPPLEMENTARY FIGURE LEGENDS

Figure S1 (A) Helicase assay testing purity of the 9-1-1 and PCNA protein preparations. **(B,C)** DNA2 cleavage activities on a dsDNA substrate and a gap substrate in the presence of the 9-1-1 complex or PCNA. Substrates used are depicted on the top of the gel with the asterisk indicating the site of the ^{32}P label.

Figure S2 (A) The loci studies are shown on the map of chromosome V and VI. **(B)** Analyses of 3'ssDNA accumulation following telomere uncapping in *cdc13-1 cdc15-2 bar1* strains. **(C)** Analyses of 5'ssDNA accumulation following telomere uncapping in *cdc13-1 cdc15-2 bar1* strains. **(D)** Analyses of DNA amount by qPCR at the indicated loci in *cdc13-1 cdc15-2 bar1* background strains. *PAC2* is a control locus located 400kb away from the right arm of chromosome V. The data and error bars plotted in B are means and standard deviations from two independent experiments, and the data and error bars in C,D are the group means and standard deviations from individual samples measured in triplicate.

Figure S3 (A) Analyses of 3'ssDNA accumulation in *cdc13-1 cdc15-2 bar1* background strains at 32ºC. The data and error bars are the means and standard deviations from individual samples measured in triplicate. **(B)** Comparison of relevant genotypes from Figure 4 and 5.

Figure S4 (A) Co-immunoprecipitation experiment to detect interaction between 9-1-1 and Dna2/Sgs1/Exo1 following *cdc13-1* induced telomere uncapping at 36°C. Protein extract from cells expressing (+) or not expressing (-) the indicated epitope tagged proteins were subjected to immunoprecipitation with anti-HA (left panel) or anti-Myc (right panel) antibodies, before probing with anti-HA (top panels) or anti-Myc (bottom panels) antibodies. **(B)** ChIP analyses of Dna2-Myc binding to *DUG1* and *PDA1* following telomere uncapping. **(C)** ChIP analyses of Mec3-Myc and Ddc1-Myc binding to *PDA1* following telomere uncapping in *cdc13-1 cdc15-2 bar1* strains. The data and error bars plotted in B and C are means and standard deviations from individual samples measured in triplicate.

Figure S5 The yeast two-hybrid system used *cdc13-1* reporter strains to test for protein interactions between Rad17, Mec3, Ddc1, Dna2, Sgs1 and Exo1. The genes of interest were fused to the Gal4 activator domain (left) or the Gal4 DNA binding domain (top) to observe interaction. Interactions were analysed using *HIS3, ADE2* and cyclo-hexamide (CHX) reporter genes at the temperatures indicated. Boxes highlight panels presented in Fig. 6B.

Table S1 and S2 Yeast strains used in the study.

Table S3 Oligonucleotides used for making substrates for nuclease assays.

Table S4 Oligonucleotides used for making substrates for two-hybrid experiments.

SUPPLEMENTARY METHOD

Helicase Assays: Five fmol of experimental substrate was incubated with 250, 500 fmol of either 9-1-1 complex or PCNA, in a reaction volume of 20 µl, on ice for 25 min. The reaction buffer contained 50 mM Tris-HCl (pH 7.5), 25 mM NaCl, 2 mM dithiothreitol, 0.25 mg/ml bovine serum albumin, 4 mM $MgCl_2$, and 8 mM ATP. Lane 6 containing 100 fmol of DNA2 alone contained 2mM $MgCl_2$ in the reaction buffer to minimize the nuclease activity of the protein. The reactions were terminated using 6X helicase dye (50 mM EDTA, 0.9% SDS, 0.125% bromphenol blue, 0.125% xylene cyanole, 30% glycerol). After termination, samples were loaded on a pre-run 5% native polyacrylamide gel and resolved by electrophoresis for 1.5 h at 150 V. Helicase products are indicated on the gel.
